# Subpicosecond Spectroscopic Ellipsometry of the Photoinduced Phase Transition in VO_2_ Thin Films

**DOI:** 10.1021/acsphotonics.4c01414

**Published:** 2024-10-10

**Authors:** Yael Gutiérrez, Saúl Vázquez-Miranda, Shirly Espinoza, Krishna Khakurel, Mateusz Rebarz, Zhen Zhang, José M. Saiz, Shriram Ramanathan, Sébastien Cueff

**Affiliations:** † Departamento de Física Aplicada, Universidad de Cantabria, Avenida de los Castros, s/n, 39005 Santander, Spain; ‡ ICMATE, CNR, Istituto di Chimica della Materia Condensata e delle Tecnologie per l’Energia, C.so Stati Uniti 4, 35127 Padova, Italy; § ELI Beamlines Facility, The Extreme Light Infrastructure ERIC, Za Radnicí 835, 25241 Dolní Břežany, Czech Republic; ∥ School of Materials Engineering, Purdue University, West Lafayette, Indiana 47907, United States; ⊥ Ecole Centrale de Lyon, CNRS, INSA Lyon, Université Claude Bernard Lyon 1, CPE Lyon, CNRS, INL, UMR5270, Université Lyon, 69130 Ecully, France

**Keywords:** insulator-to-metal transition, vanadium dioxide, ultrafast, pump−probe spectroscopy, spectroscopic ellipsometry

## Abstract

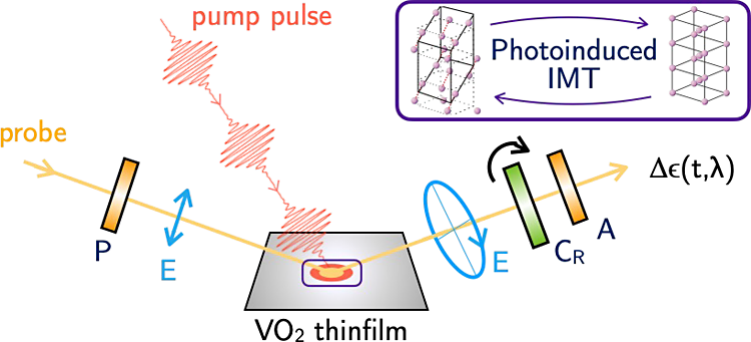

We report the first application of broadband time-resolved pump–probe ellipsometry to study the ultrafast dynamics of the photoinduced insulator-to-metal transition (IMT) in vanadium dioxide (VO_2_) thin films driven by 35 fs laser pulses. This novel technique enables the direct measurement of the time-resolved evolution of the complex pseudodielectric function of VO_2_ during the IMT. We have identified distinct thermal and nonthermal dynamics in the photoinduced IMT, which critically depends on the pump wavelength and fluence, while providing a detailed temporal and spectral phase map. A comparison of the pseudodielectric function of the VO_2_ thin film during thermally and photoinduced phase transitions reveals that the primary differences in the IMT pathways occur within the first picosecond after the pump, driven by nonequilibrium dynamics in this ultrafast time scale. The ultrafast spectroscopic ellipsometry introduced in this work offers a complementary probe to study phase changes in condensed matter and emerging photonic device materials.

## Introduction

Vanadium dioxide, VO_2_, is a correlated-electron material exhibiting an insulator-to-metal transition (IMT). The transition from an insulating to a metallic state is the result of the collapse of the band gap and formation of a metallic band structure at the IMT temperature, causing drastic changes in the electrical and optical properties of the material. The IMT is accompanied by a structural phase transition (SPT). Below *T* = 68 °C, VO_2_ has monoclinc *M* crystal symmetry with a semiconducting band structure with a band gap of 0.6 eV.^1^ At the IMT temperature of *T* ≈ 68 °C, VO_2_ undergoes a first-order phase transition to the high temperature rutile *R* phase. These simultaneous changes of both the electronic structure and crystal lattice make the phase change a rather interesting topic of study across physical sciences and engineering.^2^ Although early demonstrations of the IMT were thermally induced, it has been demonstrated that this process can also be driven optically^3^ or electrically.^4,5^

Based on this phenomenon, a plethora of devices with reconfigurable optical responses have been proposed in the literature, modulated by either thermal, electrical, and optical stimuli.^2^ Among all these devices, of special importance are those related to vanadium dioxide waveguides to be incorporated as on-chip components for photonic integrated circuits aiming to exploit the benefits of light for communication and data processing at the nanoscale.^6−14^ In this field, all-optical switching has been proposed as one of the most promising switching schemes. As the IMT can be triggered in subpicosencond time scales,^10−13^ it is possible to perform terabits per second (Tbps) on-chip optical switching as recently demonstrated.^13^ Nevertheless, the ultrafast optical switching in VO_2_ can also be relevant for other applications such as ultrafast wavefront control^2,15,16^ or image processing^17^ through VO_2_ metasurfaces.

Given the potential technological importance of the photoinduced IMT in VO_2_, its understanding and the ability to control it are crucial. The photoinduced IMT has a nonthermal basis and therefore occurs on subpicosecond time scales. The evolution and dynamics of this photoinduced IMT have been studied using a wide variety of ultrafast time-resolved techniques (e.g., electron diffraction,^3,18,19^ differential transmittance/reflectance,^11,20,21^ photoemission,^22,23^ etc.) in order to establish the ultrafast electron and lattice dynamics. In these time-resolved experiments, a strong pump pulse is used to excite the system, while a secondary pulse is used to probe a specific property as a function of the time delay between these two pulses. These experiments have demonstrated that during the photoinduced phase transition, the IMT and the SPT do not occur congruently,^24^ and metallic transient phases could appear during the process.^20,25−29^ An extensive and comprehensive review of the up-to-date knowledge of the mechanism behind the ultrafast IMT has been provided by Wegkamp and Sthäler.^23^

Among time-resolved spectroscopy techniques, broadband time-resolved spectroscopic ellipsometry^30^ is a powerful tool for determining the nature of ultrafast dynamics in complex materials and for the study of optically induced phase transitions.^31^ With this technique, changes in the complex dielectric function can be measured spectrally as a function of time after pump pulse excitation. Unlike single-wavelength time-resolved reflectance/transmittance measurements^11,20,21^—both techniques widely used to study the laser-induced IMT in VO_2_—time-resolved ellipsometry offers the additional possibility of extracting the parametrization of the complex dielectric function over a broad spectral range with a temporal resolution of a few femtoseconds. This emerging time-resolved technique has already been successfully applied to obtain the transient dielectric function of semiconductors and to study the optical properties of such materials after the photoexcitation of carriers,^32−36^ as well as to study the photoinduced insulator-to-metal transition in perovskite thin films.^37^

**Figure 1:**
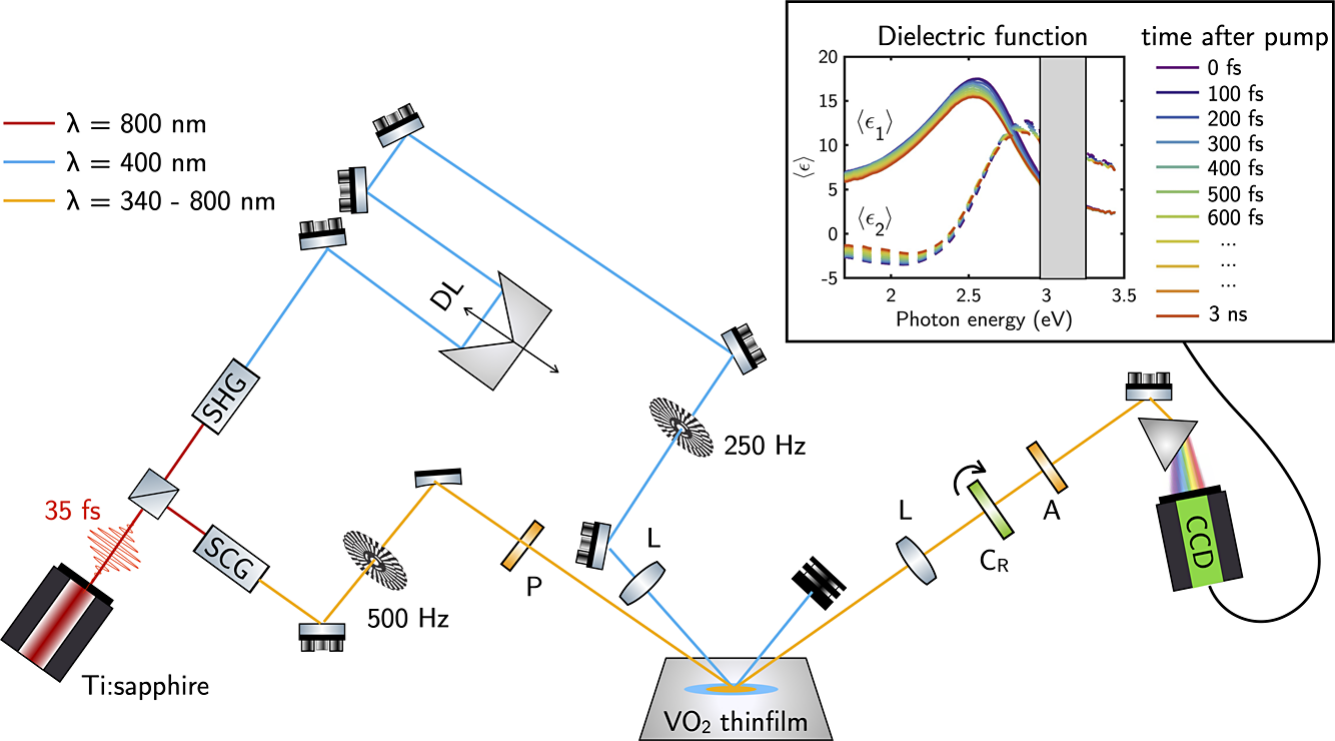
Sketch of the experimental setup for the pump–probe time-resolved spectroscopic ellipsometer. SHG, second harmonic generation for the pump-beam; SCG, supercontinuum white light generation for the probe-beam; DL, delay line; L, lens; P, polarizer; C_R_ rotating compensator; A, analyzer. The repetition rate of the pump is 250 Hz. This technique allows us to capture the complex pseudodielectric function of the VO_2_ thin film as a function of time after the pump, with a temporal resolution of 100 fs, over a broad spectral range from 1.7 to 3.5 eV.

In this work, we exploit the novel technique of broadband time-resolved pump–probe spectroscopic ellipsometry to capture, with 100 fs resolution, the spectral changes in the complex pseudodielectric function of VO_2_ thin films during the photoinduced insulator-to-metal transition, as schematized in Figure 1. For the first time to our knowledge, we compare the complex pseudodielectric function of a VO_2_ thin film across the IMT driven both thermally and by ultrashort laser pulses. By comparing these two processes, we identified that the primary differences between these pathways occur within the first picosecond after the pump. These differences are attributed to nonequilibrium dynamics. Such ultrafast dynamics have garnered significant attention, as numerous studies^20,25−29^ have reported the emergence of transient crystallographic phases in these ultrafast time scales. Additionally, the transient changes in the ellipsometric features are used to obtain characteristics of the photoinduced phase transition such as rise/decay times or threshold fluences to better understand the dynamics of the photoinduced IMT. Moreover, it should be noted that the time-resolved broadband changes in the ellipsometric parameters measured with this technique can be used to fit independently the time-dependent complex pseudodielectric function for any defined pump parameters, thus providing fundamental data sets essential for modeling ultrafast reconfigurable VO_2_ optical devices.^38−41^

## Methods

### Thin Film Fabrication

VO_2_ thin films (25 nm thickness) were deposited on quartz substrates by magnetron sputtering. The thin films exhibit a polycrystalline structure, as confirmed by X-ray diffraction (XRD) analysis. A V_2_O_5_ target was sputtered at a power of 100 W of radio frequency. During deposition, the substrate was maintained at 750 °C; the chamber pressure was kept at 5 mTorr with flowing of 49.5 sccm Ar and 0.5 sccm 10% Ar-balanced oxygen gases. The thin film XRD was collected in transmission mode using a tube X-ray operated at Copper K-α corresponding to a wavelength of 1.5406 Å.

### Temperature-Dependent Spectroscopic Ellipsometry

The complex dielectric function of the VO_2_ thin film is analyzed by using a variable-angle spectroscopic ellipsometer (UVISEL, Horiba Jobin-Yvon). The incident broadband light source (xenon lamp) is polarized at 45°. To study temperature-dependent optical properties, measurements are conducted over a temperature range of 30 to 85 °C using a digitally controlled heating stage (Linkam THMSEL350 V). The measured complex pseudodielectric function ⟨ϵ⟩ = ⟨ϵ_1_⟩ + *i*⟨ϵ_2_⟩ of the samples was taken at an angle of incidence of 65° over a spectral range of 0.75–5.00 eV. The pseudodielectric function represents a dielectric function obtained directly from the measured values ellipsometric parameters (Ψ, Δ) and is calculated from an optical model that assumes a semi-infinite flat substrate. This magnitude allows for the comparison of different materials or films under similar measurement conditions, serving as a baseline for comparative studies. Additionally, it is useful for identifying changes in material properties driven by the temperature or ultrashort laser pulses. In our experiements, since no change in the optical properties of the substrate is expected, any observed variations in the pseudodielectric function can be attributed primarily to the VO_2_ film. The complex dielectric function ϵ = ϵ_1_ + *i*ϵ_2_ of the thin film at different temperatures can be derived by fitting to a realistic multilayer air-VO_2_-quartz model of the experimental data.

### Time-Resolved Pump–Probe Spectroscopic Ellipsometry

The transient pseudodielectric function was recorded in air using a broadband femtosecond *polarizer-sample-compensator-analyzer* spectroscopic ellipsometer, as described in Richter et al.^30^ and schematized in Figure 1. For these experiments, we based the system on the titanium sapphire laser Coherent Astrella, with 800 nm fundamental wavelength that generates a supercontinuum white light pulse in a CaF_2_ window and is later filtered by an FGS900-A Thorlabs bandpass filter. The resulting 1.7–3.9 eV broadband pulse was used as the probe pulse with an angle of incidence of 65°. The sample was excited by a pump pulse of 800 nm (1.55 eV) or by its second harmonic corresponding to 400 nm (3.1 eV); its polarization was adjusted by a half waveplate to be *p-*polarized on the sample (i.e., VO_2_ thin film), and its angle of incidence was set at 70°, as collinear as possible to the probe pulse. The pump pulse repetition rate was set to 250 Hz to ensure that the film had ample time (4 ms) to fully relax between pulses.^42^ Both pump and probe pulses are set to overlap, with the pump pulse having a spot size bigger than the probe to ensure uniform excitation across the probed area. A variable delay line between the pump- and probe beams allows for time-resolved measurements. The data reduction needed for the determination of the pseudo-dielectric was done following the Mueller-Matrix formalism, a chirp correction,^30^ and by weighting of the measurements to a steady-state spectrum (Ψ and Δ spectra of the sample before the pump–probe experiment) measured at an EP4 ParkSystem ellipsometer. As a result of the measurement process, we obtain the complex pseudodielectric function of the VO_2_ thin film as a function of time after the pump, with a temporal resolution of 100 fs, over a broad spectral range from 1.7 to 3.5 eV. The raw data files from these measurements are available on Zenodo.^43^

## Results

We compare the VO_2_ thin film optical behavior across the IMT triggered thermally (static) and by ultra short laser pulses (dynamic) to provide a comprehensive analysis of this process.

### Thermally Driven IMT: Static Properties

**Figure 2:**
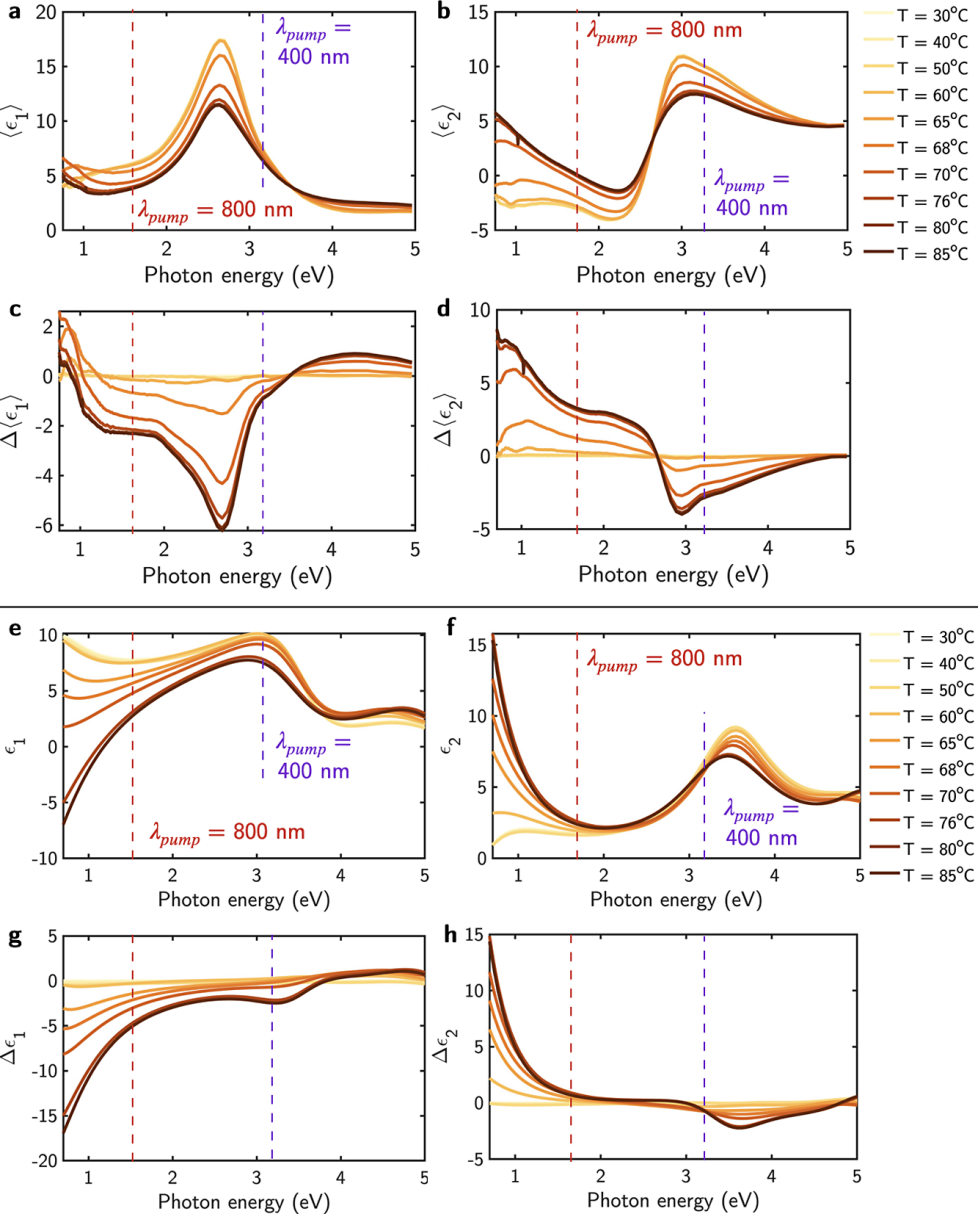
(a) Real and (b) imaginary parts of the pseudodielectric function ⟨ϵ_1_⟩ and ⟨ϵ_2_⟩ as a function of the temperature of the VO_2_ thin film. Changes in the (c) real and (d) imaginary parts of the pseudodielectric function Δ⟨ϵ_1_⟩ and Δ⟨ϵ_2_⟩ as a function of the temperature taking the ⟨ϵ⟩ at room temperature as reference. (e) Real and (f) imaginary part of the dielectric function ϵ_1_ and ϵ_2_ of the VO_2_ thin film. Changes in the (g) real and (h) imaginary parts of the pseudodielectric function Δϵ_1_ and Δϵ_2_ as a function of the temperature taking the ϵ at room temperature as reference. Vertical lines indicate the pump wavelengths λ_pump_ used in the time-resolved ellipsometry experiments.

Figure 2a,b shows the pseudodielectric function ⟨ϵ⟩ = ⟨ϵ_1_⟩ + *i*⟨ϵ_2_⟩ of a 25 nm VO_2_ thin film deposited on quartz measured with spectroscopic ellipsometry at an angle of incidence of 65° as a function of the temperature in the 30 to 85 °C range. Taking the pseudodielectric function at room temperature (RT) as reference, the changes in Δ⟨ϵ_1_⟩ and Δ⟨ϵ_2_⟩ are obtained as a function of the temperature of the film set by a heating stage and are shown in Figure 2c,d. Therefore, Δ⟨ϵ_1_⟩ and Δ⟨ϵ_2_⟩ at the different temperatures *T* are defined as Δ⟨ϵ_1_⟩ = ⟨ϵ_1_⟩_*T*_ – ⟨ϵ_1_⟩_RT_ and Δ⟨ϵ_2_⟩ = ⟨ϵ_2_⟩_*T*_ – ⟨ϵ_2_⟩_RT_. As shown in Figure 2c,d, the values of Δ⟨ϵ_1_⟩ and Δ⟨ϵ_2_⟩ are negligible for temperatures *T* ≤ 60 °C. At temperatures *T* > 68 °C, the VO_2_ thin film is driven across the IMT, and the Δ⟨ϵ_1_⟩ and Δ⟨ϵ_2_⟩ increase until reaching a maximum difference at *T* = 80 °C.^14,44^

To further investigate the optical properties of the thin film, we extract its complex dielectric function by fitting ϵ = ϵ_1_ + *i*ϵ_2_ to a physically consistent optical model. This model consists of a system composed of air-VO_2_-quartz stacked planar layers. Figure 2e,f shows the dielectric function of VO_2_ as a function of the temperature. These results align well with the literature, providing a reliable baseline for comparison.^2,44^ It can be observed how, as the thin film is heated, ϵ_1_ turns negative, and simultaneously, ϵ_2_ increases. Variation in the dielectric function with respect to the values obtained at RT is shown in Figure 2g,h. Modifications as large as Δϵ = ϵ_*T*_ – ϵ_RT_ ≈ 15 in the near-IR are reached across the IMT at *T* > 68 °C. The change from ϵ_1_ > 0 to ϵ_1_ < 0 in this spectral range is indicative of the insulator (dielectric) to metal transition in the thin film. Taking this into consideration, we establish a metallic upper bound for Δ⟨ϵ_1_⟩ = −1.95 at 2 eV as indicative of metallic behavior (ϵ_1_ < 0) in the dielectric function of the VO_2_ thin film. This reference value is taken from Δ⟨ϵ_1_⟩ at 2 eV and *T* = 70 °C, just above the IMT temperature. Therefore, observed values of Δ⟨ϵ_1_⟩ < −1.95 can be associated with the metallic phase of VO_2_.

In all plots of Figure 2, vertical lines indicate the pump wavelengths λ_pump_ of 400 (3.1 eV) and 800 nm (1.55 eV) that are used in the time-resolved pump–probe ellipsometry experiments described in the following section.

### Photoinduced IMT: Dynamic Properties

The broadband transient pseudodielectric function Δ⟨ϵ⟩ of the 25 nm VO_2_ thin film was probed in the 1.7 to 3.5 eV range as a function of the fluence and wavelength of the 35 fs pump pulses. The effects of two different pump wavelengths λ_pump_ are compared in this study: λ_pump_ = 400 nm (3.1 eV) and λ_pump_ = 800 nm (1.55 eV). The latter, the most often used λ_pump_ in pump–probe time-resolved spectroscopic studies of VO_2_,^3,18,20,21^ can be used as reference for comparison with other studies. In particular, it has been reported very recently that the IMT and SPT are decoupled at the subpicosecond time scale upon 800 nm photoexcitation.^24^ The photoexcitation produces holes in the *d*_∥_ valence band, which initiates the dilation of V–V pairs and the increase of twisting angles, driving the structural phase transition from *M*-VO_2_ to *R*-VO_2_. In the case of 400 nm, there is a lack of both experimental and theoretical studies at this pump wavelength; therefore, the excitation mechanism behind the structural *M*-to-*R* transition is worth investigating to develop a general framework for photoinduced phase transitions.

For both λ_pump_, the penetration depth δ_p_ in the insulating state is greater than the thickness of the film, i.e., δ_p_ = 68 nm for λ_pump_ = 400 nm and δ_p_ = 196 nm for λ_pump_ = 800 nm. This ensures that the entire thickness of the VO_2_ film is uniformly excited by the pump pulse. The pump fluence *F*_pump_ used in this study varied between 1.536 and 3.072 mJ/cm^2^ for λ_pump_ = 800 nm and from 3.058 to 7.646 mJ/cm^2^ for λ_pump_ = 400 nm.

**Figure 3:**
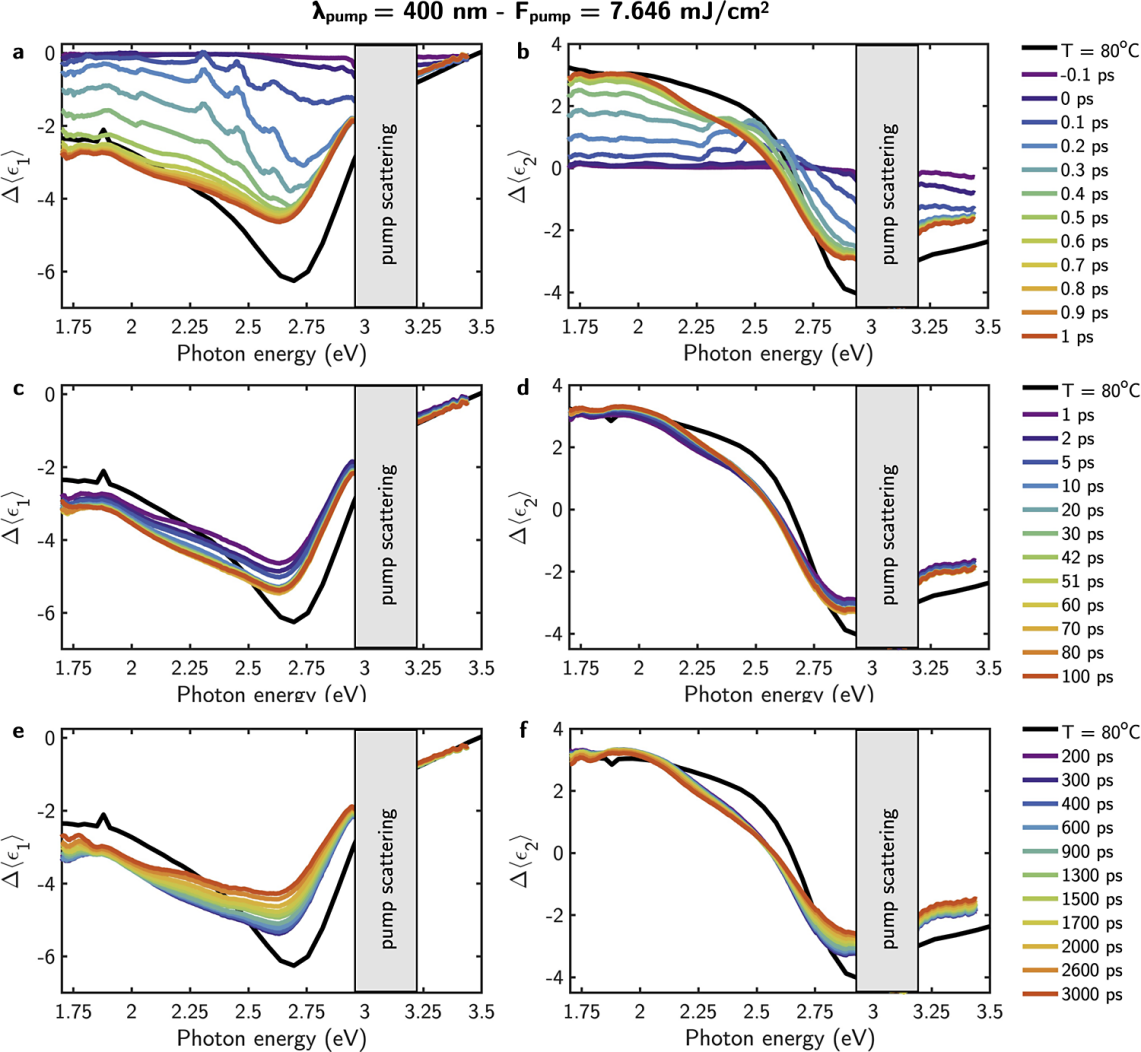
Transient (a, c, e) real and (b, d, f) imaginary part of the pseudodielectric function in the time delay intervals of [−0.1, 1], [1, 100], and [200, 3000] ps. The pump characteristics are λ_pump_ = 400 nm and power = 7.646 mJ/cm^2^. As a reference, these plots also show the maximum modulation of Δ⟨ϵ_1_⟩ and Δ⟨ϵ_2_⟩ achieved thermally by heating the thin film to *T* = 80 °C.

**Figure 4:**
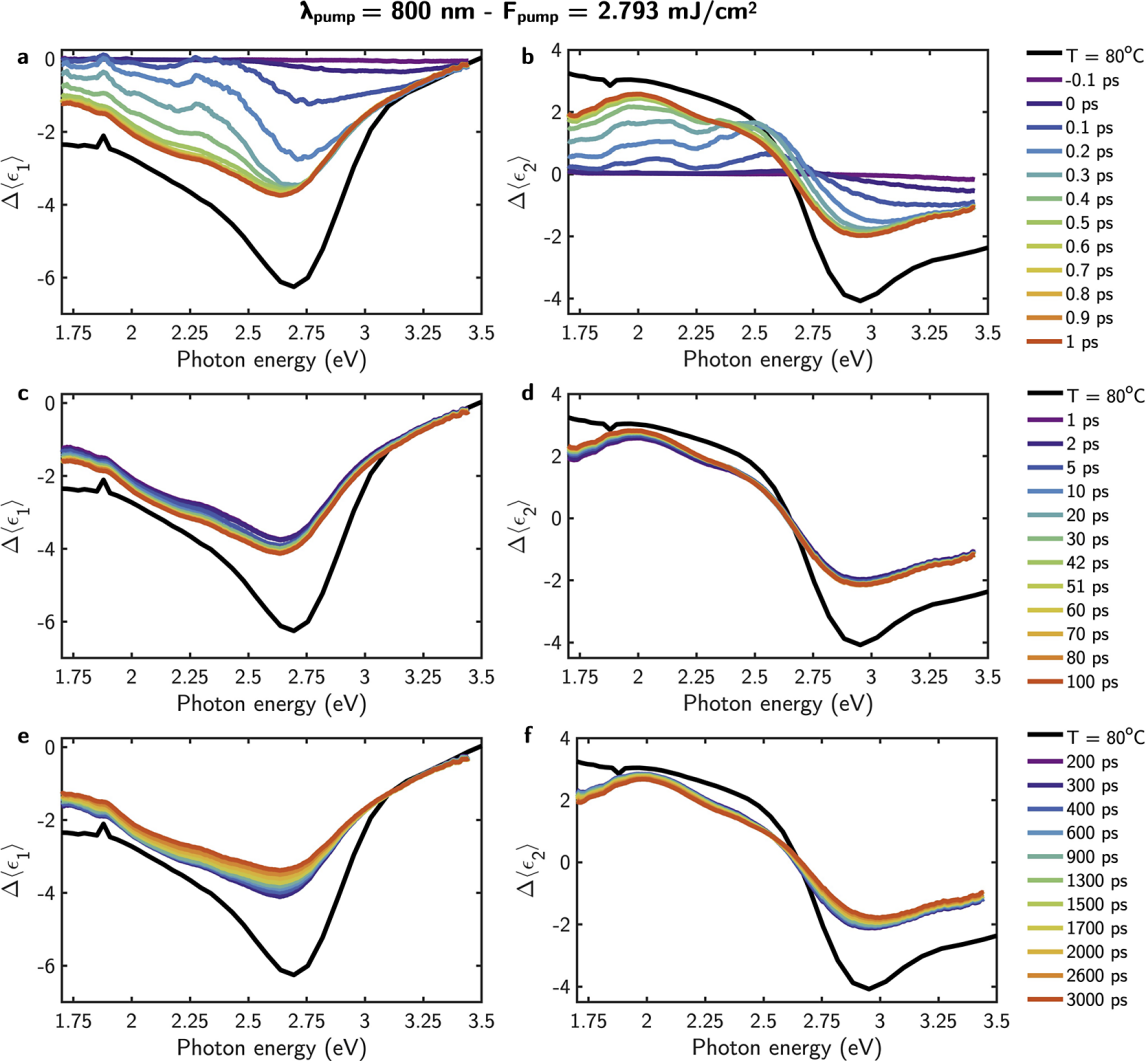
Transient (a, c, e) real and (b, d, f) imaginary parts of the pseudodielectric function in the time delay intervals of [−0.1, 1], [1, 100], and [200, 3000] ps. The pump characteristics are λ_pump_ = 800 nm and power = 2.793 mJ/cm^2^. As a reference, these plots also show the maximum modulation of Δ⟨ϵ_1_⟩ and Δ⟨ϵ_2_⟩ achieved thermally by heating the thin film to *T* = 80 °C.

Figures 3 and 4 illustrate the spectral values of Δ⟨ϵ_1_⟩ and Δ⟨ϵ_2_⟩ at time delays ranging from 0 to 3000 ps, following excitation with pump pulses of λ_pump_ = 400 nm with *F*_pump_ = 7.646 mJ/cm^2^, and λ_pump_ = 800 with *F*_pump_ = 2.793 mJ/cm^2^, respectively. These plots show the results obtained with the maximum *F*_pump_ values employed in the experiment, which result in the strongest variation of the optical properties of the VO_2_ film. Nevertheless, corresponding plots for the remaining *F*_pump_ values used in this study are provided in the Supporting Information. Additionally, as a reference, these plots also show the maximum Δ⟨ϵ_1_⟩ and Δ⟨ϵ_2_⟩ achieved thermally by heating the thin film to *T* = 80 °C. Figures 3 and 4 highlight the similarity in the spectral line-shape between the photoinduced Δ⟨ϵ_1_⟩ and Δ⟨ϵ_2_⟩ and the thermally induced variation, as presented in Figure 2c,d.

**Figure 5:**
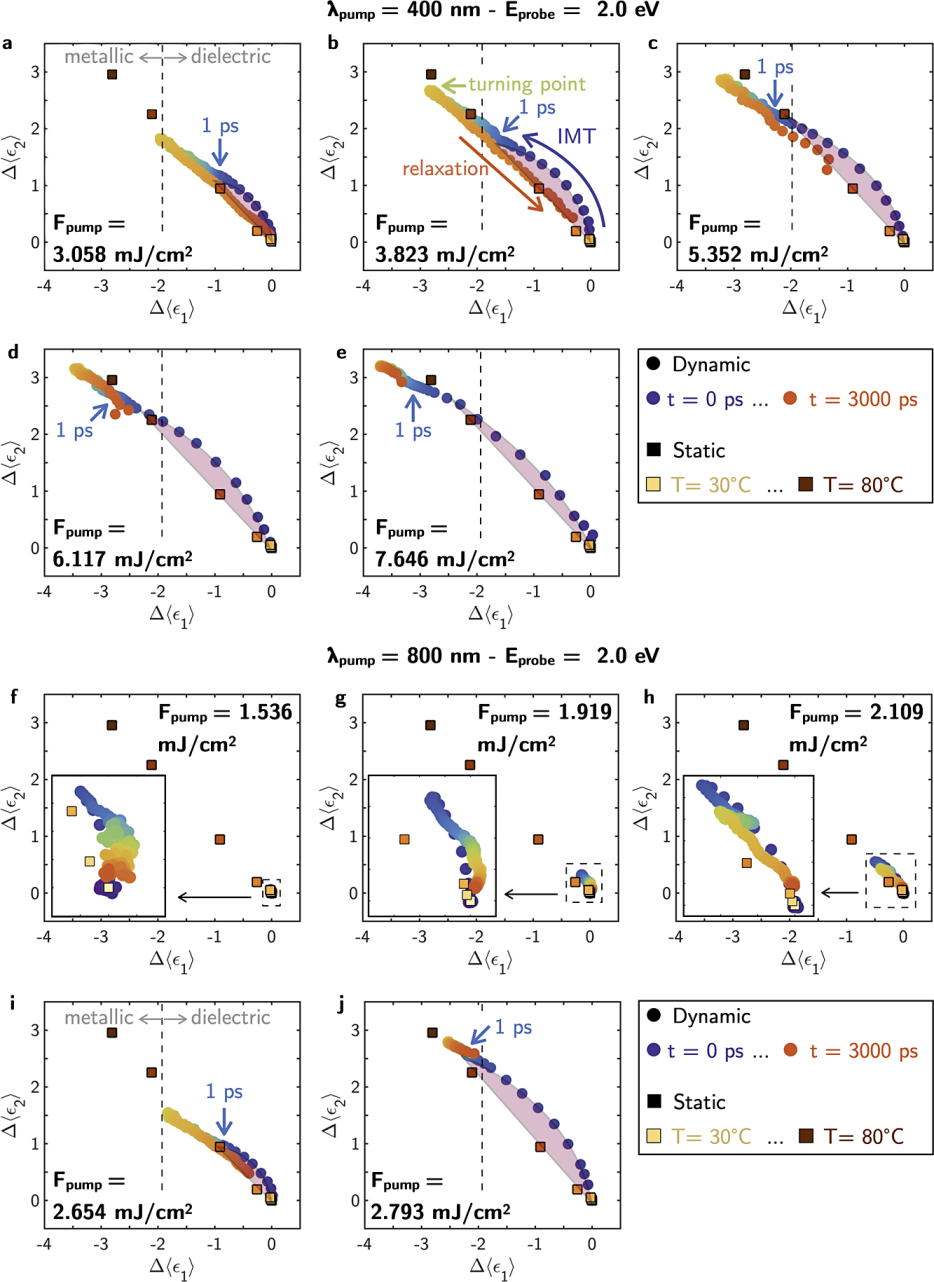
Dynamic Δ⟨ϵ_2_⟩ vs Δ⟨ϵ_1_⟩ as a function of delay time *t* at *E*_probe_ = 2 eV for pump wavelengths (a–e) λ_pump_ = 400 nm and (f–j) λ_pump_ = 800 nm. Each plot corresponds to a different pump fluence *F*_pump_. For comparison, it is shown the static Δ⟨ϵ_2_⟩ vs Δ⟨ϵ_1_⟩ at *E*_probe_ = 2 eV as a function of the temperature of the film *T*. The vertical dashed line indicates the Δ⟨ϵ_1_⟩ = −1.95 limit, which allows us to identify metallic and dielectric behavior in the dielectric function of the film. The purple shadowed regions highlights the difference between the thermal and photoinduced IMT paths.

In order to explore how the changes in Δ⟨ϵ_1_⟩ and Δ⟨ϵ_2_⟩ synchronize with each other as a function of the *F*_pump_, Figure 5 shows evolution of Δ⟨ϵ_1_⟩ vs Δ⟨ϵ_2_⟩ monitored at a photon energies *E*_probe_ = 2 eV and at time delays following the excitation with pump pulses of λ_pump_ = 400 nm and λ_pump_ = 800 nm. Each plot corresponds to a different fluence of the pump pulse. For comparison, the static evolution of these same parameters as a function of the temperature of the film is also shown. Additionally, the vertical dashed line indicates the Δ⟨ϵ_1_⟩ = −1.95 limit, which allows us to identify metallic and dielectric behavior in the dielectric function of the film. Independently of λ_pump_, some general trends can be identified:

1. The trajectory of Δ⟨ϵ_2_⟩ vs Δ⟨ϵ_1_⟩ during the photoinduced IMT closely aligns with that observed during the static thermally induced transition. However, it is interesting to distinguish two trends in the photoexcitation process of the IMT. Within the first picosecond following the pump, the trajectory of Δ⟨ϵ_2_⟩ vs Δ⟨ϵ_1_⟩ displays the most pronounced deviation from the thermal path. This first picosencond is of great interest because of the dominance of nonequilibrium dynamics that leads to novel physical phenomena. The discrepancy between nonthermal and thermal paths, highlighted with a purple shadow in Figure 5, may arise from the dominance of nonequilibrium dynamics in which strong electronic excitation, according to Cocker et al.,^29^ results in the photoexcited electron-assisted nucleation of the rutile phase (nonthermal process). Other authors have also reported the emergence of short-lived (few hundreds of picoseconds) transient phases after the pump.^20,25−29^ After 1 ps, the photoinduced and thermal paths achieve good overlap until reaching a turning point in the ⟨ϵ_2_⟩ vs ⟨ϵ_1_⟩ trajectory. During this time, the energy of the laser pulse is converted into lattice heating, and thus, the comparatively slower growth of the metallic rutile phase.^29^ At higher delay times than the turning point, the relaxation back to the insulating state takes place. During this relaxation process, which is mediated by the cooling of the film, the trajectory of Δ⟨ϵ_2_⟩ vs Δ⟨ϵ_1_⟩ closely follows the thermal transition path. This described behavior can be clearly seen in Figure 5b, where each process (IMT and relaxation) and characteristic point (1 ps and turning point) are indicated.
2. This behavior in the values of Δ⟨ϵ_1_⟩ and Δ⟨ϵ_2_⟩, which do not follow the same trajectory during excitation and relaxation, leads to an hysteresis effect in the transient pseudodielectric function.
3. The amplitude of Δ⟨ϵ_1_⟩ and Δ⟨ϵ_2_⟩ increases with the fluence of the pump independently of λ_pump_. In the case of λ_pump_ = 400 nm, for all the analyzed *F*_pump_, the metallic pseudodielectric function in the film is achieved. On the contrary, in the case of λ_pump_ = 800 nm, for *F*_pump_ lower than 2.654 mJ/cm^2^, the limit in Δ⟨ϵ_1_⟩ for the metallic behavior is not reached. Therefore, in the case of λ_pump_ = 800 nm, a fluence threshold for the photoinduced IMT can be defined. On the contrary, for λ_pump_ = 400 nm, all the measurements are performed above that threshold.
4. Increasing *F*_pump_ not only amplifies the modulation in the optical response of the film but also extends the recovery time to the initial insulating state. This behavior is consistent with the literature.^9^

**Figure 6:**
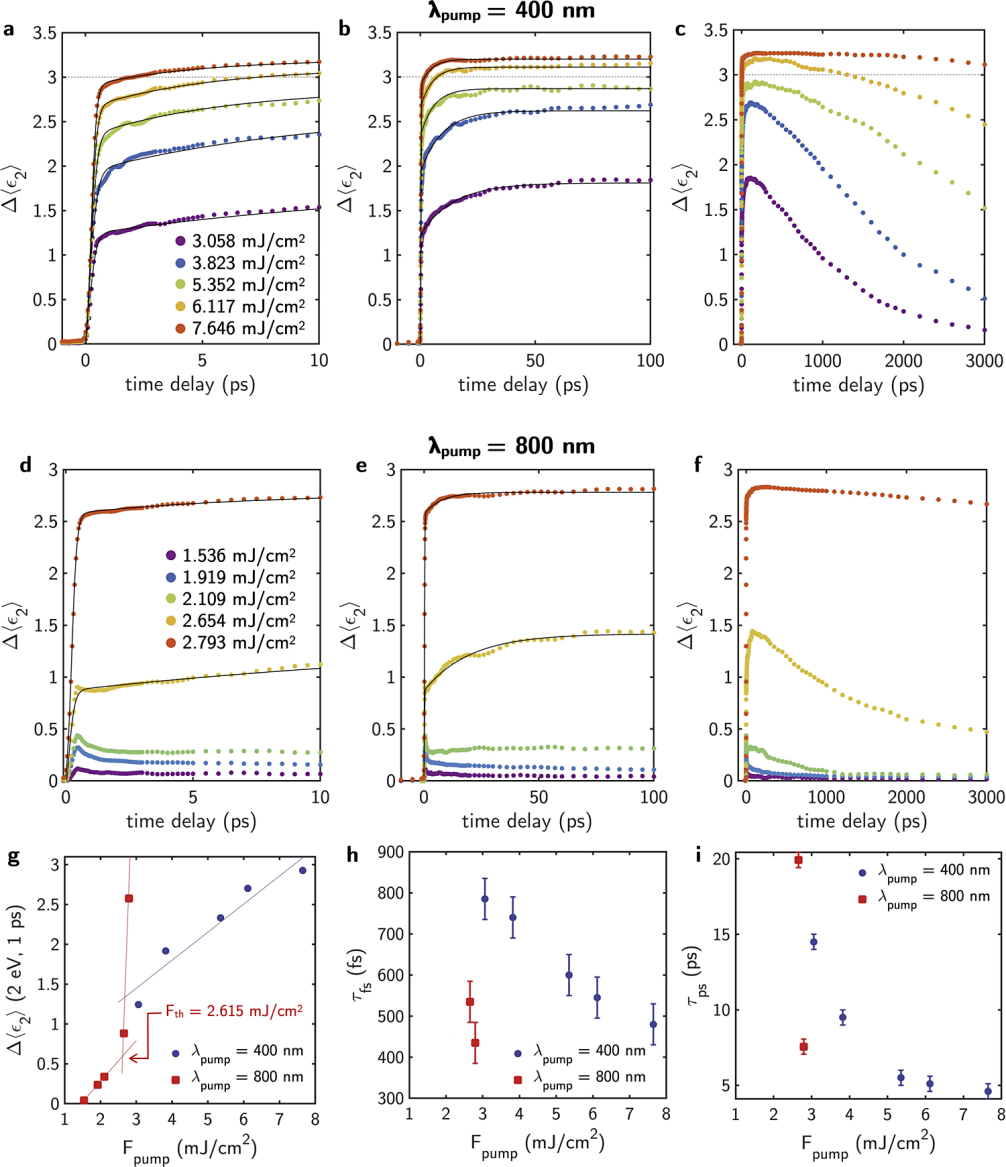
Time-resolved Δ⟨ϵ_2_⟩ at 2 eV of the VO_2_ thin film at different time scales for pump wavelengths of (a–c) λ_pump_ = 400 nm and (d–f) λ_pump_ = 800 nm. In (a–c), a horizontal dashed line indicates the Δ⟨ϵ_2_⟩ observed for the fully metallic state at 80 °C. For each λ_pump_, the photoinduced Δ⟨ϵ_2_⟩ is shown for five pump fluences. Dots indicate the measured experimental data, and the solid lines represent results of fitting the data. (g) Value of Δ⟨ϵ_2_⟩ measured at 2 eV and delay time of 1 ps as a function of the pump fluence for λ_pump_ = 400 nm and λ_pump_ = 800 nm. Rise times for the (h) short τ_fs_ and (i) long-lived τ_ps_ behavior are extracted from the fitting to the model in eq 1.

The main difference between λ_pump_ = 400 nm and λ_pump_ = 800 nm is related to the fluence dependence of Δ⟨ϵ_1_⟩ vs Δ⟨ϵ_2_⟩. In order to address this issue and provide with the photoinduced IMT temporal dynamics, Figure 6 shows the time-resolved Δ⟨ϵ_2_⟩ of the 25 nm VO_2_ thin film probed at *E*_probe_ = 2 eV after excitation by λ_pump_ 400 and 800 nm pump pulses for the five different pump fluences, as shown in Figure 5.

In order to drive the photoinduced IMT, the fluence of the pump pulse should be higher than a certain threshold *F*_th_.^20^ Below *F*_th_, no phase transition occurs, and the material remains in the insulating state. However, in this regime, some authors, based on time-dependent reflectance^25^/transmittance^26^ and THz conductivity^27−29^ measurements, have reported a transient metallic phase that decays to near initial conditions on a time scale of 2–5 ps and that results from a coherent phonon excitation.^13^ Above the threshold, only a small region of the sample is initially transformed to the metallic phase and the dynamics are slow, being governed by heat diffusion into the sample, which results in the growth of the metallic phase.^20,45^ Nevertheless, as the *F*_pump_ increases, the transition occurs more rapidly until it reaches a saturation point, suggesting that nonthermal processes can also drive the structural changes.^3^

Figure 6a–c shows the time-resolved Δ⟨ϵ_2_⟩ for a λ_pump_ = 400 nm and pumping energies between 3.058 and 7.646 mJ/cm^2^. All the curves show a similar profile consisting of a fast sharp increase of Δ⟨ϵ_2_⟩ followed by a long-lived slow rise of the signal. To investigate the process, the rise response function of Δ⟨ϵ_2_⟩ at *t* ≥ 0 was phenomenologically fitted to
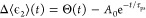
1where  represents a logistic sigmoidal function. *A*_0_, *A*_1_, α, and β are fitting parameters. The sigmoidal function describes a fast nonthermal process which is ascribed to the photoinduced nucleation of the metallic phase.^29^ The rise time τ_fs_ of this nonthermal process is defined as the time it takes for the Θ(*t*) function to go from 10 to 90% of its maximum value. The exponential term in eq 1 describes a slower process with rise time τ_ps_ ascribed to the thermally driven growth and coalescence of metal domains in the material.^29^

According to the fitting, photoinduced nucleation and growth dynamics consistent with excitation above *F*_th_ are observed in Figure 6a,b. The values τ_fs_ and τ_ps_ extracted from the fitting, plotted as a function of the pump fluence in Figure 6h,i, show that both τ_fs_ and τ_ps_ decrease with increasing *F*_pump_. Therefore, as *F*_pump_ increases, the photoinduced nucleation and subsequent growth of the metallic domains occur faster. This is also visible in Figure 5a–e, which shows how the limit indicative of metallic behavior in ϵ_1_ is reached at shorter time delays for increasing *F*_pump_. Additionally, Figure 6g shows that the value of Δ⟨ϵ_2_⟩, 1 ps after the pump at 2 eV, follows a linear increase with the pump fluence, supporting an above *F*_th_ regime for all explored *F*_pump_. This linear increase of Δ⟨ϵ_2_⟩ with *F*_pump_ implies that an increasing volume fraction of the VO_2_ thin film converts into the metallic state.

Notably, at higher pump fluences, τ_ps_ stabilizes at its lowest value, which is accompanied by the highest values of Δ⟨ϵ_2_⟩ at 1 ps, which indicates that the transition to the metallic state is dominated by nonthermal mechanisms, reaching the above *F*_th_ saturation regime. This is particularly evident for pump fluences of 6.117 and 7.646 mJ/cm^2^. As shown in Figure 6a,b, for these pump fluences, Δ⟨ϵ_2_⟩ reaches values higher than those obtained thermally after approximately 2 and 10 ps, respectively. Additionally, Figure 5d,e demonstrates that the photoinduced IMT path surpasses the thermal path in about 1 ps, supporting the dominating nonthermal behavior associated with the fast photoexcited-electron-assisted nucleation of the rutile phase.

The response of the film was monitored until 3000 ps (3 ns) after the pump, as shown in Figure 6c. This allowed us to analyze recovery times of the initial insulating state. It is possible to observe that the recovery time increases with the pump fluence. In all the analyzed cases, the time for the full recovery of the monoclinic phase is higher than 3 ns. For instance, the recovery of 80% of the signal is achieved after 2 and 3 ns for *F*_pump_ = 3.058 and 3.823 mJ/cm^2^. For the rest of the analyzed *F*_pump_ at λ_pump_ = 400 nm, these values exceed our 3 ns measurement range.

Figure 6d–f shows the time-resolved Δ⟨ϵ_2_⟩ for λ_pump_ = 800 nm and pump fluences between 1.536 and 2.793 mJ/cm^2^. In this case, measurements with higher *F*_pump_ than ≈2.793 mJ/cm^2^ produced irreversible damage to the film. In the studied range of *F*_pump_, two types of dynamics can be seen. On the one hand, for pump fluences ≥2.654 mJ/cm^2^, the profile Δ⟨ϵ_2_⟩ consists of a fast sharp increase of Δ⟨ϵ_2_⟩ followed by a long-lived slowly rising similar to that observed for λ_pump_ = 400 nm in the *F*_pump_ range 3.058 to 7.646 mJ/cm^2^. However, for λ_pump_ = 800 nm, this regime is observed in a narrower pump fluence window. The dynamics were fitted to eq 1 to extract the values of τ_fs_ and τ_ps_. As in the case of λ_pump_ = 400 nm, both τ_fs_ and τ_ps_ decrease with an increase in pump fluence. It is noteworthy that values of τ_fs_ in the case of λ_pump_ = 800 nm are lower, indicating a faster photoinduced nucleation of the metallic phase in the VO_2_ film. The values of τ_ps_ show a stronger variation with the pumping fluence than in the case of λ_pump_ = 400 nm, suggesting that the growth velocity has a stronger dependence on the pump fluence. In Figure 6f, which shows the dynamics up to 3000 ps after the pump, it can be seen how the recovery times to the full monoclinic phase for pump fluences ≥2.654 mJ/cm^2^ also exceed 3 ns.

For pump fluences ≤2.109 mJ/cm^2^, the dynamics in Figure 6d–f show a different behavior. The pump fluence threshold in the change of dynamics has been estimated to *F*_th_ ≈ 2.615 mJ/cm^2^ by the change in the slope of the dependence of Δ⟨ϵ_2_⟩ 1 ps after the pump at 2 eV as a function of the pump fluence, as shown in Figure 6g. This value agrees well in order of magnitude with other values reported in the literature, although the absolute value appears to be dependent on the thickness of the film with a threshold fluence higher for thicker films (6.1 mJ/cm^2^ for 200 nm VO_2_ thin film^20^).

Below the threshold fluence, the Δ⟨ϵ_2_⟩ profile exhibits rapid peak switching with an elevated background after the switching is completed. Similar changes in transient reflectance and transmittance measurements have been associated with a transient metallic state.^13,25,26^ However, in the spectral range analyzed here, for these *F*_pump_, the pseudodielectric function does not exhibit a metallic behavior, as shown in Figure 5f–h. Interestingly, the recovery time of the Δ⟨ϵ_2_⟩ in the ≤2.109 mJ/cm^2^ pump fluence range is lower than in the above *F*_th_ regime. In particular, after 1 ns, between 80 and 85% of the signal is recovered. Therefore, in the below *F*_th_ regime, although we can achieve a lower variation of the pseudodielectric function of the film, the recovery times are fast, making this regime interesting to consider further for optical communications applications.^11,13^

Finally, it should be noted that the lifetime of VO_2_ switching performance at high pulse repetition frequency has been identified as an important issue.^13^ In the case of photoinduced IMT, very recently, it has been reported the record of 10^7^ cycles without degradation in 3 μm long and 40 nm thick VO_2_ patches on Si waveguides using pulses of 100 ns and wavelength 1550 nm with a frequency of 10 kHz.^46^ In our experiments, for both λ_pump_ = 400 nm and λ_pump_ = 800 nm, each of the presented time-resolved pump–probe ellipsometric measurements requires 3 × 10^7^ pump pulses. This number is derived from the product of the measurement duration and the pump repetition rate. Therefore, we demonstrate here an endurance lifetime of at least 3 × 10^7^ cycles in a 25 nm VO_2_ thin film induced by 35 fs pulses with a repetition rate of 250 Hz for λ_pump_ and *F*_pump_ used in this study.

## Conclusions

**Figure 7:**
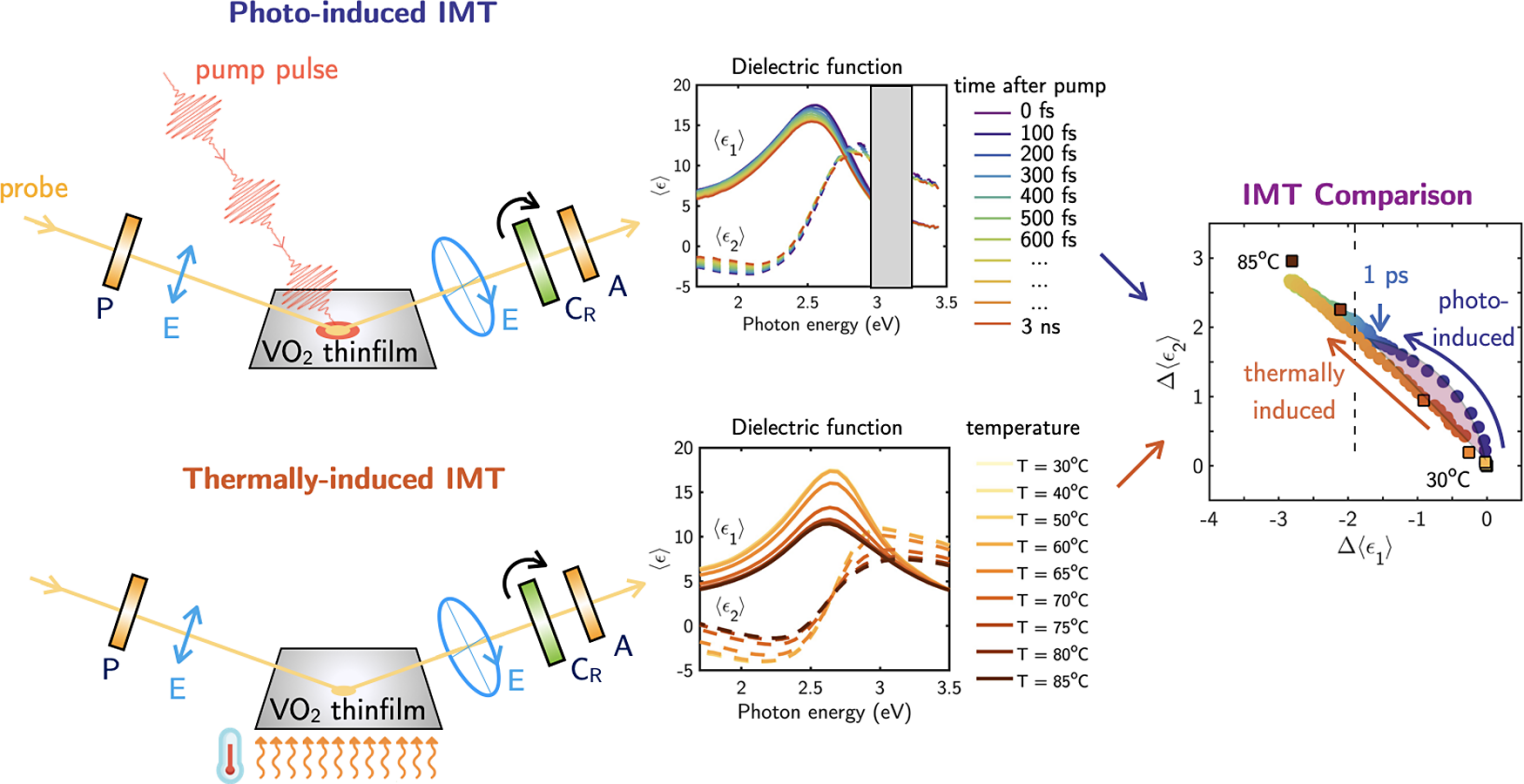
Schematic summary of the ellipsometric measurements performed in this work to capture the pseudodielectric function of a VO_2_ thin film across the photoinduced and thermally induced IMT. In the case of the photoinduced IMT, the pseudodielectric function is obtained as a function of the time after pump pulse. For the thermally induced IMT, the pseudodielectric function is measured as a function of temperature. The comparison between both IMT pathways reveals that the most pronounced discrepancies occur within the first picosecond after the pump, as highlighted by the purple shadow.

This study presents the first broadband time-resolved pump–probe spectroscopic ellipsometry measurements of the photoinduced IMT in VO_2_ films. By employing this technique, we capture the complex pseudodielectric function of the VO_2_ thin film during the photoinduced IMT with a temporal resolution of 100 fs across a broad spectral range from 1.7 to 3.5 eV. The changes in the optical properties during the photoinduced IMT have been directly compared to those during the thermally activated IMT, offering a comprehensive spectral and temporal mapping of the photoinduced IMT, as schematized in Figure 7. A direct comparison of the complex pseudodielectric function values across the IMT for thermal and laser-induced pathways reveals that the most pronounced discrepancies occur within the first picosecond after the pump driven by nonequilibrium dynamics in this ultrafast scale (shadowed region in Figure 7).

In our experiments, by employing 35 fs pump pulses at two distinct wavelengths (λ_pump_ = 400 and 800 nm) with varied fluences, we discerned diverse thermal and nonthermal dynamics governing the excitation and relaxation of the photoinduced IMT. We identified two types of behavior depending on the fluence of the pump according to what has been reported in the literature. Below a certain fluence threshold, no metallic phase is observed, although a transient in the pseudodielectric function is observed with a relaxing time (≈1 ns). Above the threshold, the observed dynamics are consistent with an initial fast (below 1 ps) nonthermal photoinduced nucleation of the metallic phase, a subsequent slow growth of the metallic domains governed by thermal diffusion, and relatively long relaxing times (>3 ns) to the insulating phase.

Additionally, under our experimental conditions, we report a cycle lifetime of at least 3 × 10^7^ cycles, aligning well with record photoinduced IMT cyclability reported by Seoane et al.^46^ These findings not only deepen our comprehension of the ultrafast dynamics intrinsic to the IMT in VO_2_ films but also underscore the utility of time-resolved pump–probe spectroscopic ellipsometry as an effective tool for investigating phase transitions in strongly correlated materials. Furthermore, time-resolved pump–probe ellipsometry offers an avenue for independent evaluation of complex dielectric function with defined pump parameters that is essential to guide the applications of phase change materials in emerging photonic device technologies.

## Data Availability

The data underlying this study are openly available at Zenodo.^43^

## Supporting Information Available

Transient pseudodielectric function in the time delay intervals of [−0.1, 1], [1, 100], and [500, 3000] ps for λ_pump_ = 400 nm and *F*_pump_ = 3.058, 3.823, 5.352, and 6.117 mJ/cm^2^; transient pseudodielectric function in the time delay intervals of [−0.1, 1], [1, 100], and [500, 3000] ps for λ_pump_ = 800 nm and *F*_pump_ = 1.536, 1.919, 2.109, and 2.654 mJ/cm^2^ (PDF)
