# Supplementary material for: Subpicosecond Spectroscopic Ellipsometry of the Photoinduced Phase Transition in VO2 Thin Films
Source: ACS Photonics. 2024 Oct 10;11(11):4883–93. doi: 10.1021/acsphotonics.4c01414 (PMC11583299; doi:10.1021/acsphotonics.4c01414)
Supplement: Supplementary file 1 — ph4c01414_si_001.pdf [file ph4c01414_si_001.pdf]

Supplementary Information for: Sub-picosecond spectroscopic ellipsometry of photo-induced phase transition in VO2 thin films

Yael Gutiérrez,[*,†,‡] Saúl Vázquez-Miranda,[¶] Shirly Espinoza,[¶] Krishna Khakurel,[¶] Mateusz Rebarz,[¶] Zhen Zhang,[§] José M. Saiz,[†] Shriram Ramanathan,[§] and Sebastien Cueff[||]

†*Departamento de Física Aplicada, Universidad de Cantabria, Avenida de los Castros, s/n, 39005 Santander, Spain*

‡*Istituto di Chimica della Materia Condensata e delle Tecnologie per l'Energia, ICMATE, CNR, C.so Stati Uniti 4, 35127 Padova, Italy*

¶*ELI Beamlines Facility, The Extreme Light Infrastructure ERIC, Za Radnicí 835, 25241 Dolní Břežany, Czech Republic.*

§*School of Materials Engineering, Purdue University, West Lafayette, IN 47907, USA*

||*Université Lyon, Ecole Centrale de Lyon, CNRS, INSA Lyon, Université Claude Bernard Lyon 1, CPE Lyon, CNRS, INL, UMR5270, 69130 Ecully, France*

E-mail: gvelay@unican.es

1. Transient dielectric function $\langle\Delta\epsilon\rangle$ for λ_{pump} = 400 nm

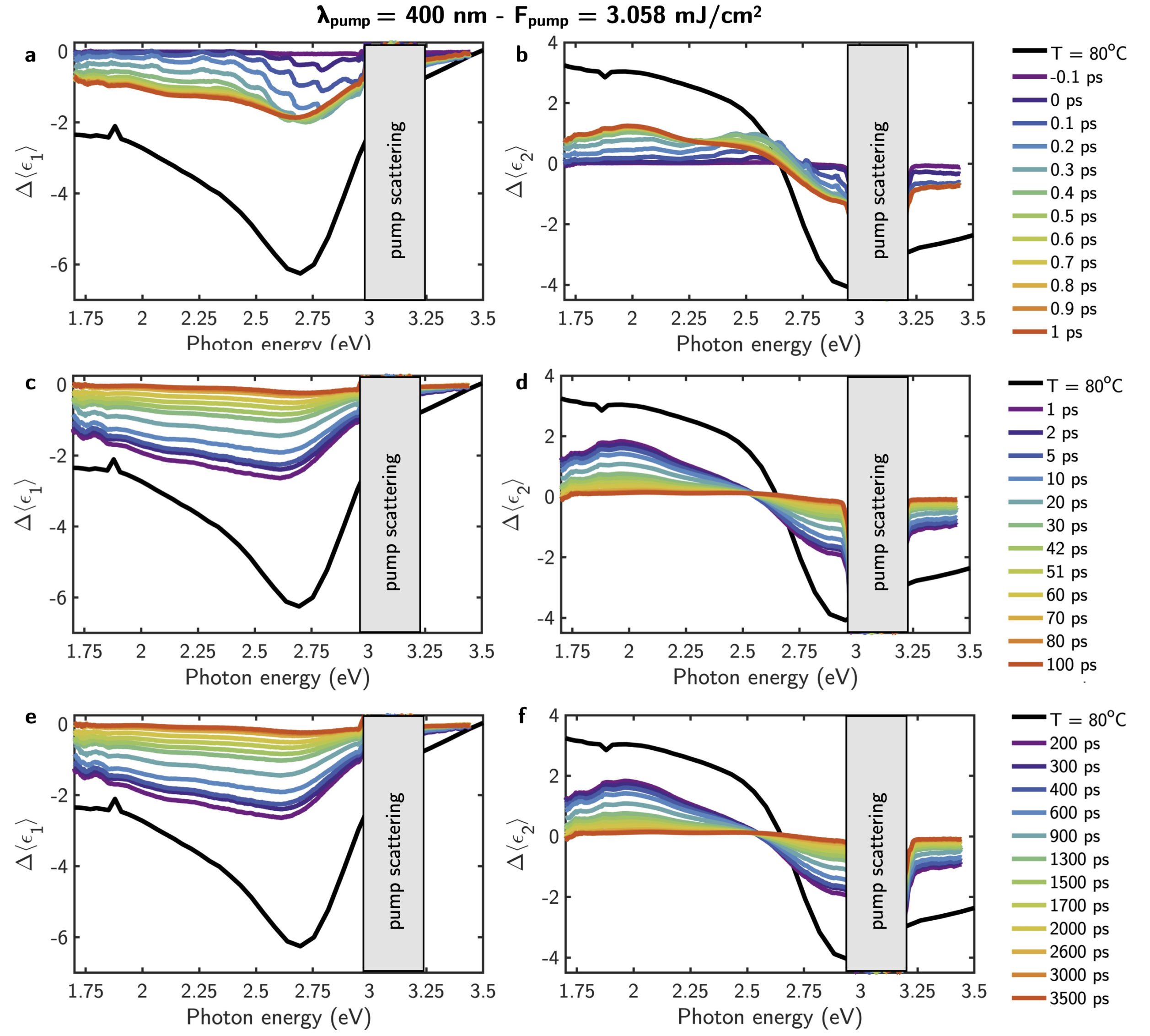

Figure S1: Transient (a,c,e) real and (b,d,f) imaginary part of the pseudo dielectric function in the time delay intervals of [-0.1, 1] ps, [1, 100] ps and [500, 3000] ps. The pump characteristics are λ_{pump} = 400 nm and F_{pump} = 3.058 mJ/cm^2. As a reference, these plots also show the maximum modulation of $\Delta\langle\epsilon_1\rangle$ and $\Delta\langle\epsilon_2\rangle$ achieved thermally by heating the thin film to T = 80°C.

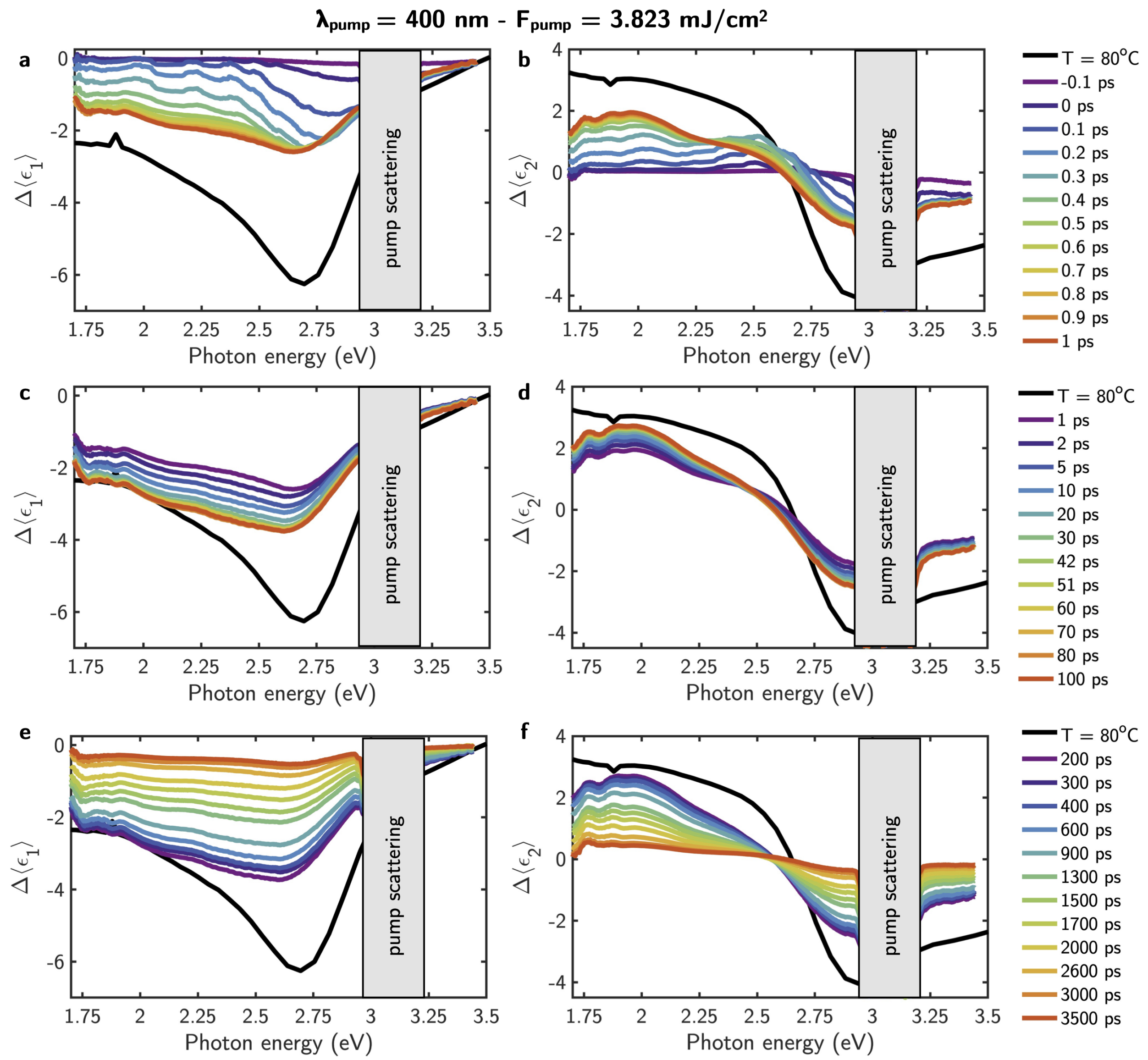

Figure S2: Transient (a,c,e) real and (b,d,f) imaginary part of the pseudo dielectric function in the time delay intervals of [-0.1, 1] ps, [1, 100] ps and [500, 3000] ps. The pump characteristics are λ_{pump} = 400 nm and F_{pump} = 3.823 mJ/cm^2. As a reference, these plots also show the maximum modulation of $\Delta\langle\epsilon_1\rangle$ and $\Delta\langle\epsilon_2\rangle$ achieved thermally by heating the thin film to T = 80°C.

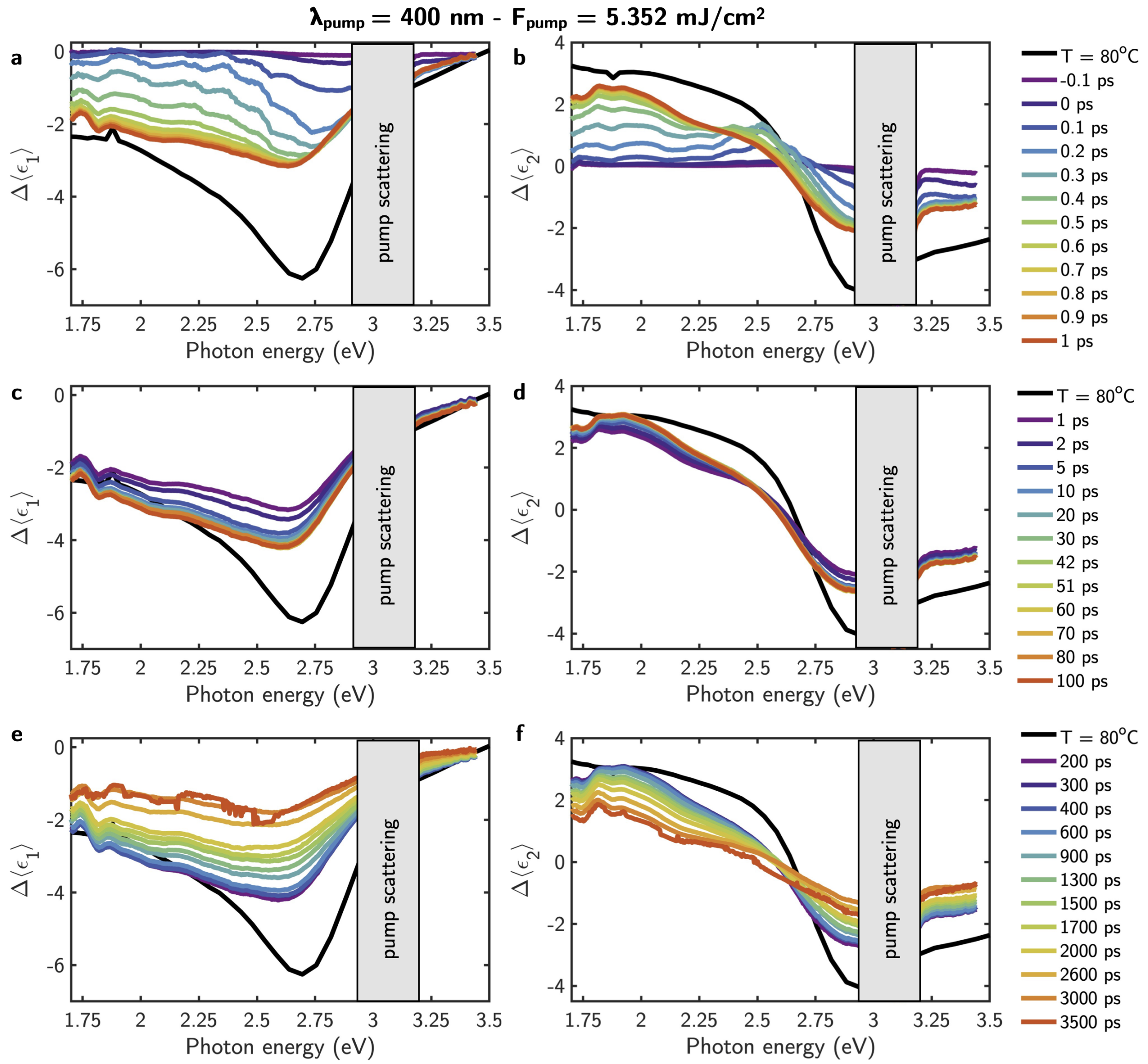

Figure S3: Transient (a,c,e) real and (b,d,f) imaginary part of the pseudo dielectric function in the time delay intervals of [-0.1, 1] ps, [1, 100] ps and [500, 3000] ps. The pump characteristics are λ_{pump} = 400 nm and F_{pump} = 5.352 mJ/cm^2. As a reference, these plots also show the maximum modulation of $\Delta\langle\epsilon_1\rangle$ and $\Delta\langle\epsilon_2\rangle$ achieved thermally by heating the thin film to T = 80°C.

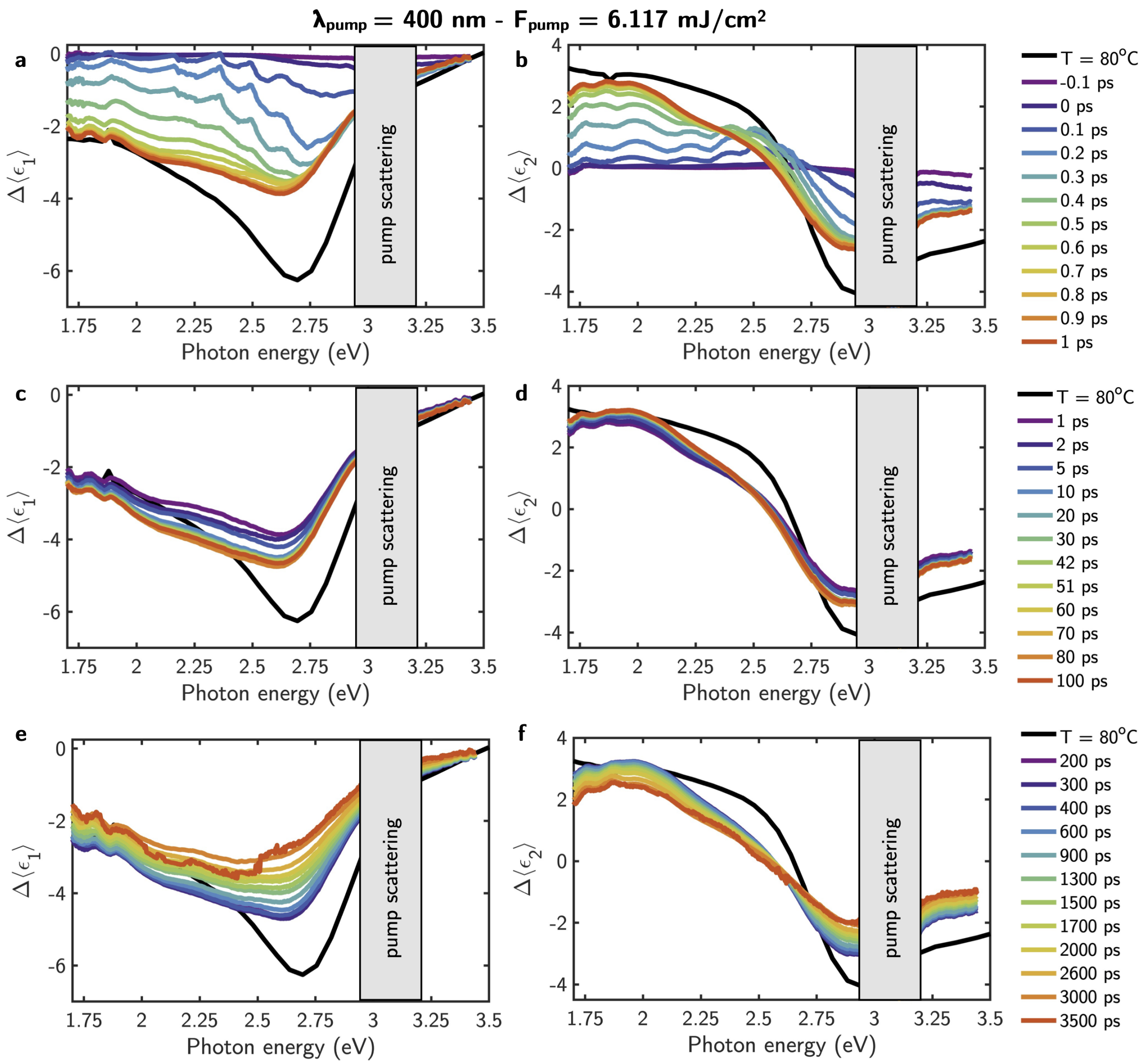

Figure S4: Transient (a,c,e) real and (b,d,f) imaginary part of the pseudo dielectric function in the time delay intervals of [-0.1, 1] ps, [1, 100] ps and [500, 3000] ps. The pump characteristics are λ_{pump} = 400 nm and F_{pump} = 6.117 mJ/cm^2. As a reference, these plots also show the maximum modulation of $\Delta\langle\epsilon_1\rangle$ and $\Delta\langle\epsilon_2\rangle$ achieved thermally by heating the thin film to T = 80°C.

2. Transient dielectric function $\langle \Delta\epsilon \rangle$ for λ_{pump} = 800 nm

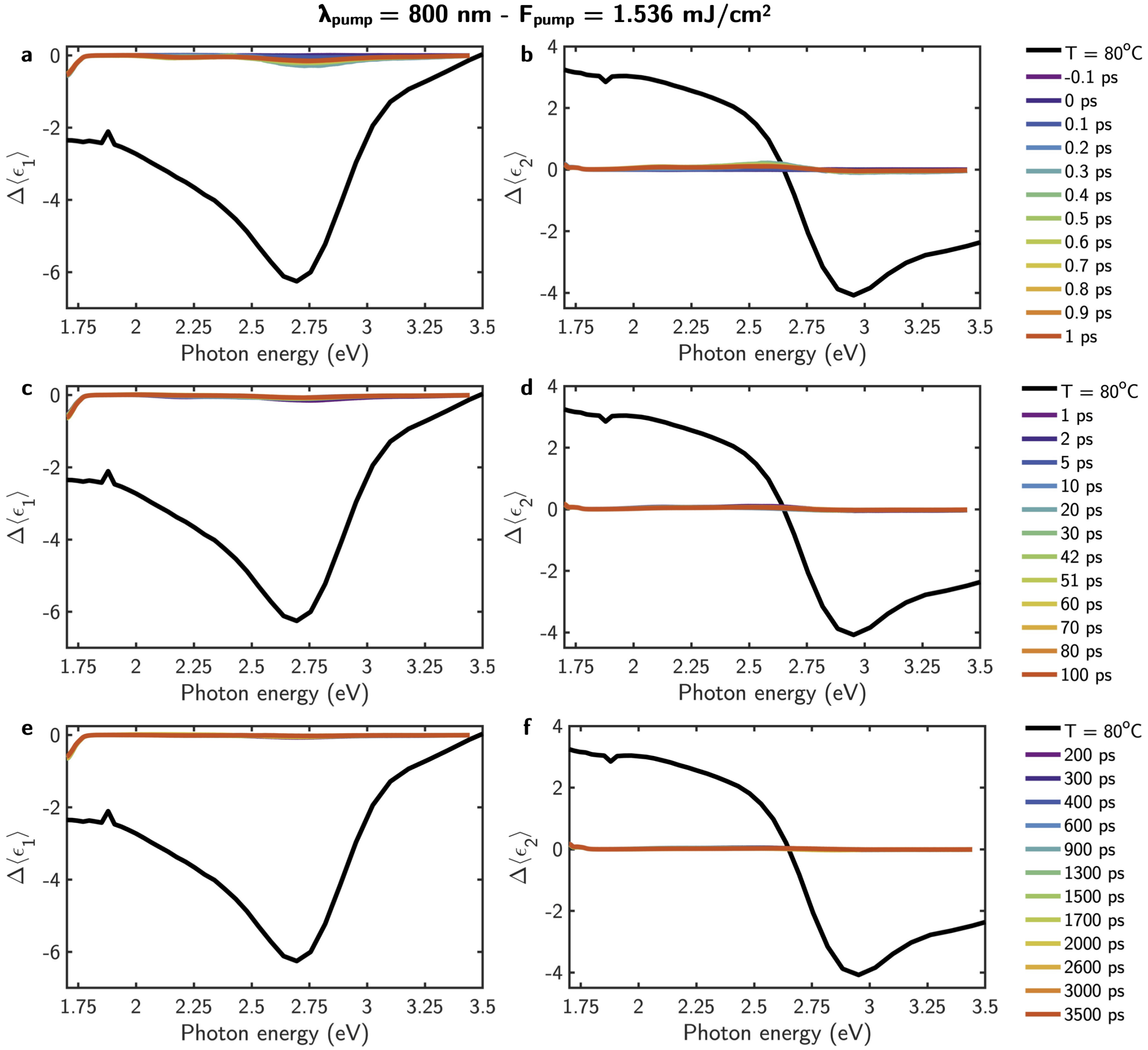

Figure S5: Transient (a,c,e) real and (b,d,f) imaginary part of the pseudo dielectric function in the time delay intervals of [-0.1, 1] ps, [1, 100] ps and [500, 3000] ps. The pump characteristics are λ_{pump} = 800 nm and F_{pump} = 1.536 mJ/cm^2. As a reference, these plots also show the maximum modulation of $\Delta\langle\epsilon_1\rangle$ and $\Delta\langle\epsilon_2\rangle$ achieved thermally by heating the thin film to T = 80°C.

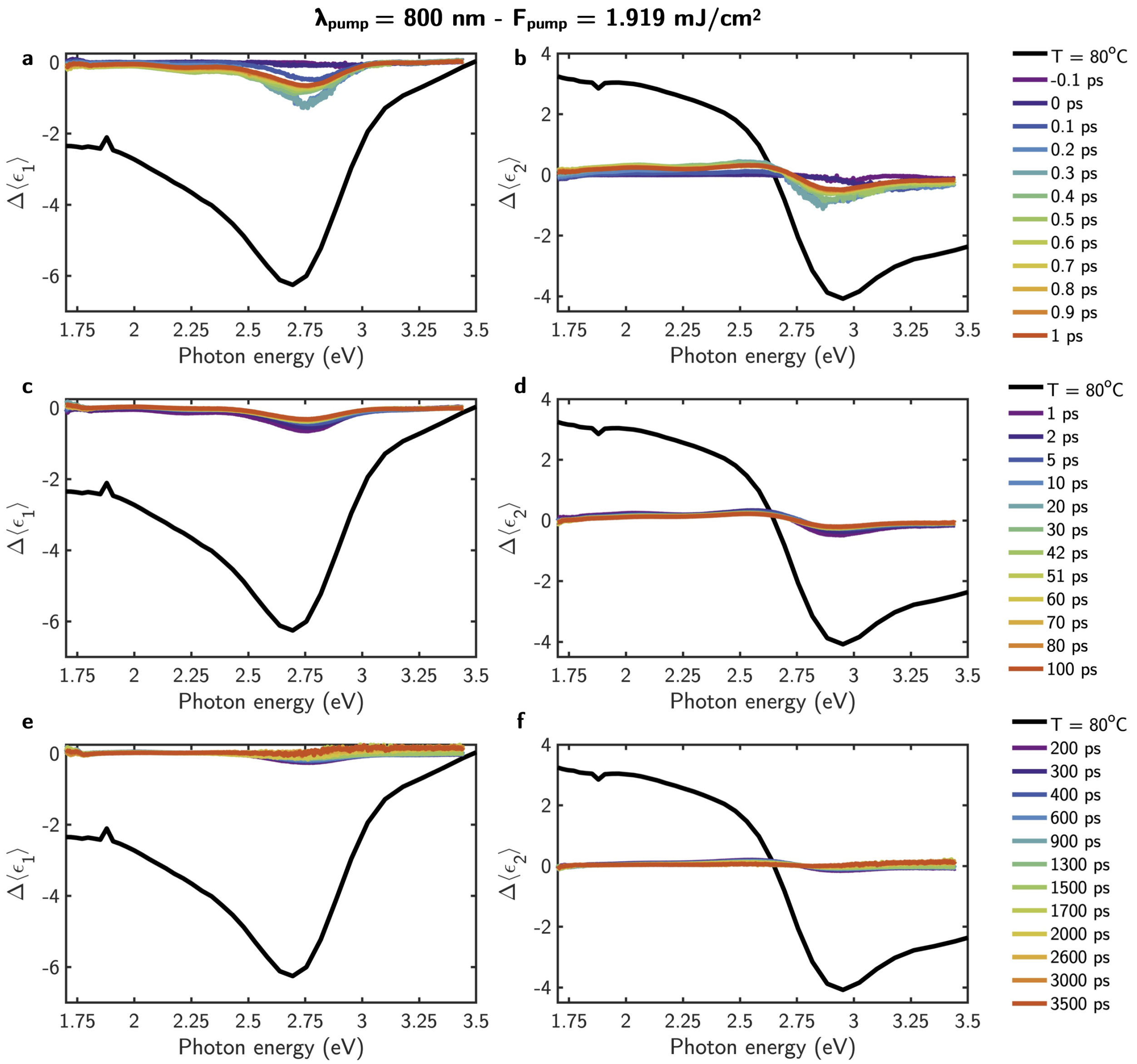

Figure S6: Transient (a,c,e) real and (b,d,f) imaginary part of the pseudo dielectric function in the time delay intervals of [-0.1, 1] ps, [1, 100] ps and [500, 3000] ps. The pump characteristics are λ_{pump} = 800 nm and F_{pump} = 1.919 mJ/cm^2. As a reference, these plots also show the maximum modulation of $\Delta\langle\epsilon_1\rangle$ and $\Delta\langle\epsilon_2\rangle$ achieved thermally by heating the thin film to T = 80°C.

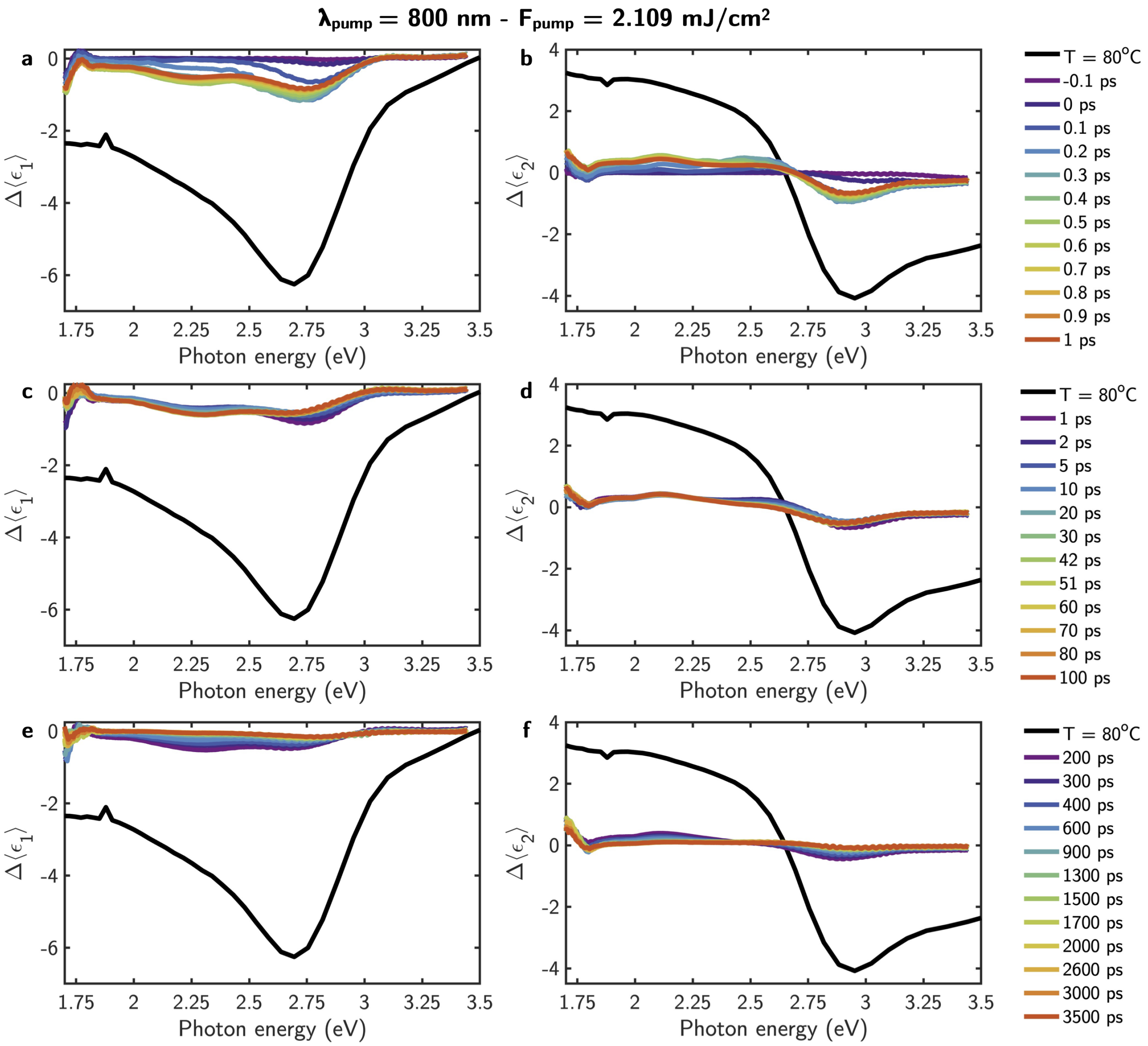

Figure S7: Transient (a,c,e) real and (b,d,f) imaginary part of the pseudo dielectric function in the time delay intervals of [-0.1, 1] ps, [1, 100] ps and [500, 3000] ps. The pump characteristics are λ_{pump} = 800 nm and F_{pump} = 2.109 mJ/cm^2. As a reference, these plots also show the maximum modulation of $\Delta\langle\epsilon_1\rangle$ and $\Delta\langle\epsilon_2\rangle$ achieved thermally by heating the thin film to T = 80°C.

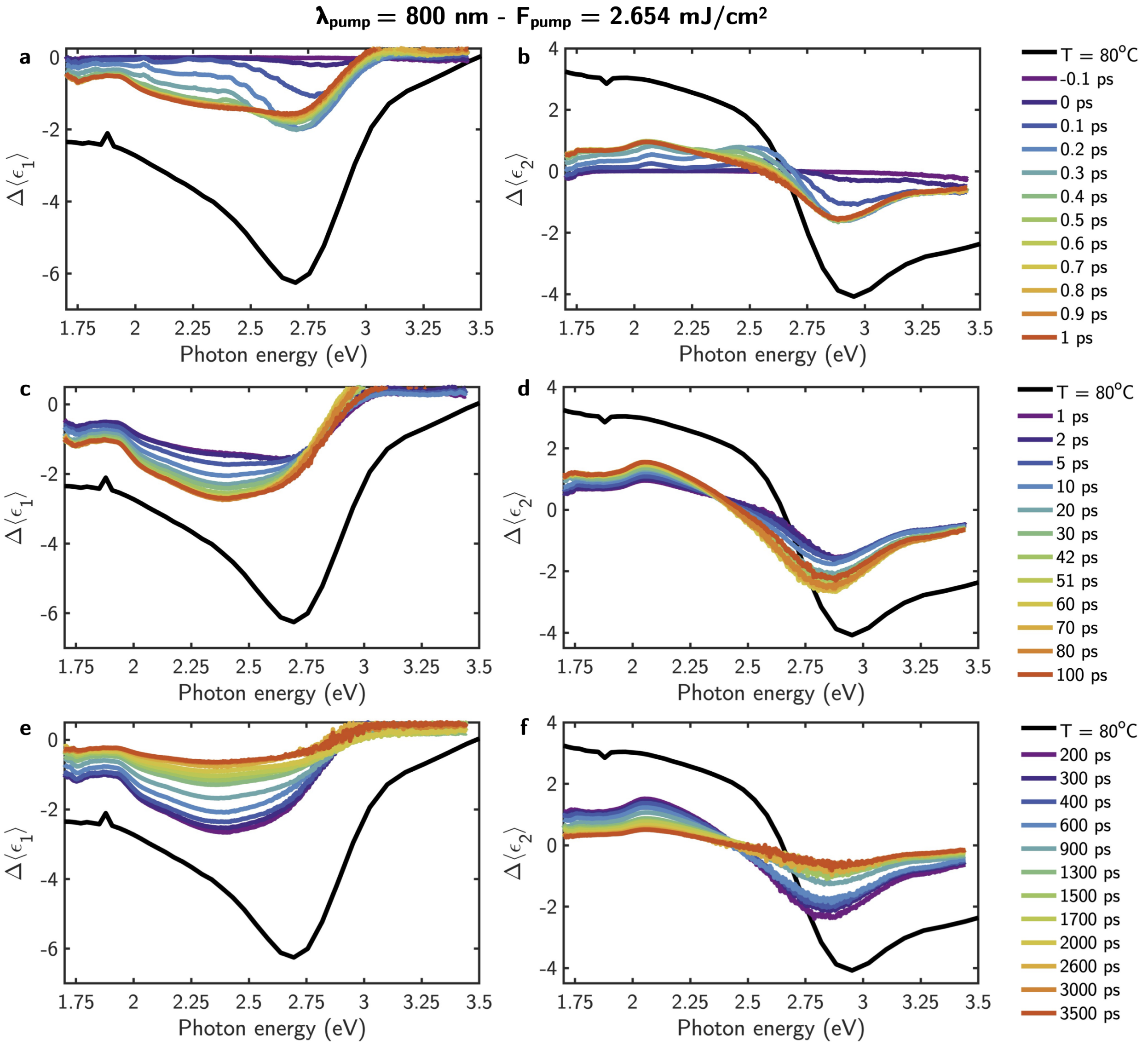

Figure S8: Transient (a,c,e) real and (b,d,f) imaginary part of the pseudo dielectric function in the time delay intervals of [-0.1, 1] ps, [1, 100] ps and [500, 3000] ps. The pump characteristics are λ_{pump} = 800 nm and F_{pump} = 2.654 mJ/cm^2. As a reference, these plots also show the maximum modulation of $\Delta\langle\epsilon_1\rangle$ and $\Delta\langle\epsilon_2\rangle$ achieved thermally by heating the thin film to T = 80°C.
